# Camel Milk Extracellular Vesicles as Functional Foods and Nutraceuticals: Bridging Dairy Science and Chronic Disease Prevention

**DOI:** 10.3390/ijms27135777

**Published:** 2026-06-26

**Authors:** Hui Yang, Yajun Xu, Rili Ge

**Affiliations:** 1 College of Medical, Qinghai University, Xining 810016, China; xuyajun@bjmu.edu.cn (Y.X.); gerili@qhu.edu.cn (R.G.); 2 School of Public Health, Peking University, Beijing 100191, China

**Keywords:** camel milk, extracellular vesicles, functional foods, nutraceuticals, chronic diseases, immunomodulation, dairy nanovesicles, gut health

## Abstract

Camel milk is increasingly recognized as a premium functional food, attributed to its rich nutraceutical compounds. Recent research has concentrated on the nanoscale extracellular vesicles derived from camel milk (CM-EVs), which exhibit distinctive properties. This review examines the methodologies for isolating and characterizing CM-EVs, alongside their potential health benefits in functional foods and nutraceuticals. CM-EVs have the capacity to safeguard functional proteins, noncoding RNAs, and bioactive lipids from degradation within the gastrointestinal tract, rendering them particularly suitable for incorporation into infant formulas, adult dietary supplements, and nutraceuticals targeting chronic inflammatory and metabolic disorders. Preclinical models indicate that CM-EVs can mitigate oxidative stress, enhance intestinal barrier integrity, and modulate gut microbiota, thereby contributing to the reduction in colonic injury and inflammation. Nonetheless, the majority of these findings are derived from laboratory and animal studies, highlighting a substantial deficiency in human clinical trials. Critical research gaps remain, necessitating further investigation into the elucidation of molecular mechanisms, assessment of long-term safety, evaluation of bioavailability, and compatibility with dairy processing techniques. This review underscores the significance of CM-EVs as bioactive food components and delineates research priorities, such as standardizing isolation methods, investigating food matrix integration, and providing translational evidence for their application in nutrition and preventive medicine.

## 1. Introduction

Camel milk (CM) has historically been an essential nutritional resource in arid regions, characterized by its higher concentrations of medium-chain fatty acids, whey proteins, and vitamin C compared to bovine milk [1]. Beyond its nutritional value, CM contains a diverse range of bioactive compounds, including specific peptides, lactic acid bacteria, and immunoglobulins, which impart antimicrobial, anti-inflammatory, antioxidant, and immunomodulatory properties [2,3]. As a result, CM is increasingly recognized as a functional food and a source of nutraceuticals—food-derived products with proven therapeutic benefits for the prevention or management of chronic diseases such as inflammatory bowel disease, metabolic syndrome, and disorders related to oxidative stress.

Recent research has concentrated on the nanoscale components present in cow’s milk, with a particular emphasis on extracellular vesicles (EVs). These membrane-bound structures, which include exosomes and microvesicles, are integral to intercellular communication. Notably, EVs derived from Camel’s milk (CM-EVs) exhibit unique compositional and functional properties that distinguish them from EVs found in other mammalian milks [4]. CM-EVs are characterized by the encapsulation of specific mRNA transcripts and proteins. Of particular interest is the reported differential impact of CM-EVs on reactive oxygen species (ROS) levels: they are capable of increasing ROS to induce apoptosis in malignant cells while simultaneously enhancing antioxidant defenses in healthy tissues [2,5]. The observed dual functionality can likely be ascribed to differences in molecular cargo. By “molecular cargo,” we denote both the intraluminal content (comprising nucleic acids, proteins, lipids, and metabolites) and the surface-associated molecules (including tetraspanins, immunomodulatory surface proteins, and glycoconjugates), as both compartments play a crucial role in modulating extracellular vesicle (EV) signaling and functional outcomes. These elements are essential considerations in the formulation of nutraceuticals designed for the prevention of chronic diseases.

Notwithstanding the challenges associated with the elevated lipid content of CM-EVs, recent advancements in isolation techniques have substantially propelled research into the potential applications of CM-EVs in the context of metabolic and immune-mediated disorders [4,6]. As naturally occurring nanocarriers, CM-EVs offer considerable advantages for nutritional and nutraceutical applications. Derived from an abundant dairy source, CM-EVs enable cost-effective and scalable extraction, exhibiting minimal immunogenicity and high biocompatibility [7,8]. The robust phospholipid bilayer of CM-EVs effectively safeguards encapsulated substances—such as noncoding RNAs, functional proteins, and bioactive lipids—from degradation in the gastrointestinal tract, thereby enhancing their bioavailability. In the field of food science, these properties render CM-EVs promising candidates for fortifying infant formulas and adult dietary supplements. Preclinical studies suggest that oral administration of CM-EVs can mitigate hypoxia-induced colonic injury by fortifying the intestinal mucosal barrier, modulating gut microbiota, and restoring metabolic homeostasis [4,9].

Despite the increasing interest in CM-EVs, the existing literature remains fragmented, revealing significant knowledge gaps regarding their specific molecular mechanisms, long-term safety profiles, and optimal integration into food matrices [10,11]. We hypothesize that CM-EVs possess unique compositional and functional properties that make them promising candidates for use as functional foods and nutraceuticals, particularly in the context of chronic disease prevention. Significantly, there have been no published human clinical trials, with the majority of findings originating from in vitro studies or rodent models. We delineate the platforms utilized for our research (PubMed, Web of Science, Scopus, and Google Scholar), as well as the search terms and Boolean operators employed (“camel milk” AND “extracellular vesicles” OR “exosomes” OR “nanovesicles”). The inclusion and exclusion criteria were set to English-language, peer-reviewed articles published up to December 2025. This review systematically examines the isolation, characterization, and biofunctional properties of camel milk-derived extracellular vesicles (CM-EVs) within the framework of functional foods and nutraceuticals. It highlights their intrinsic value as bioactive components sourced from food and delineates future research directions aimed at facilitating their translation for human health. Particular emphasis is placed on their compatibility with dairy processing and the development of evidence-based functional products for the prevention and management of chronic diseases.

## 2. Comparison and Optimization of Isolation Methods

The isolation of CM-EVs presents considerable technical challenges due to the intricate biochemical composition of CM, characterized by its high fat content (approximately 2–5%) and unique protein profile, which includes caseins, whey proteins, and immunoglobulins. These components can result in the substantial co-isolation of impurities [4,7,10]. A mechanistic rationale for the removal of casein is essential, as it constitutes a critical pre-analytical step. Casein micelles, which range in size from 50 to 500 nm, overlap with the size of EVs, necessitating their efficient removal. This is accomplished through several chemical principles: (1) During EDTA chelation, EDTA sequesters calcium ions ($Ca^{2+}$) from calcium phosphate nanoclusters that stabilize casein micelles, leading to their dissociation into soluble casein monomers. (2) During sodium citrate disruption, citrate functions both as a calcium chelator and a phosphate competitor, disrupting the colloidal calcium phosphate crosslinks within casein micelles, thereby diminishing micellar integrity. (3) Acid precipitation involves adjusting the pH to the isoelectric point of caseins (around pH 4.6), neutralizing their surface charge and causing them to aggregate and precipitate. This method is cost-effective but may risk EV aggregation or cargo leakage if not managed properly. In contrast, chelation and citrate methods avoid extreme pH levels that could harm EV integrity. Differential ultracentrifugation (UC), which separates EVs by size and density at 100,000–150,000× *g*, remains the most widely used technique [12,13].

**Figure 1:**
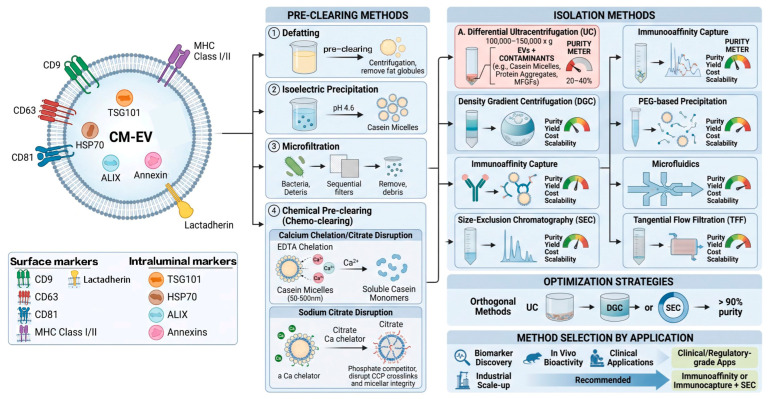
Overview of isolation methods for CM-EVs in functional food and nutraceutical applications [1,4,7,13,16,17,18]. A shows that the purity of the extract obtained by UC was relatively low, as indicated by the red coloration.

While UC can process large volumes (up to several hundred milliliters) and offers industrial scalability, it frequently co-sediments casein micelles, protein aggregates, and milk fat globule fragments, resulting in EV purity typically ranging from only 20–40% in a single UC step [14,15]. Density gradient centrifugation (DGC) separates EVs based on their buoyant density, achieving over 90% purity, but is labor-intensive, requires expertise, and is not suitable for high-throughput application due to limited sample capacity. Immunoaffinity capture uses antibodies on magnetic beads or chromatography matrices to isolate EVs with high specificity and purity, but it is costly, not scalable, and may miss marker-negative EVs. Size-exclusion chromatography (SEC) separates EVs by size using porous beads [7,16]. SEC offers excellent preservation of EV structure and activity with high purity but faces challenges with sample dilution and limited fractionation volumes. PEG-based precipitation is fast and cost-effective but results in low purity, making it suitable mainly for pilot studies. Microfluidic technologies provide rapid, automated EV isolation from small samples and show promise for dairy quality control, though they currently lack high throughput and industrial validation [7,17] (Figure 1). Refer to Table 1 for a detailed comparison of all methods evaluated across nine parameters: principle, time, yield, purity, scalability, cost, sample volume, downstream compatibility, and suitability for CM.

Optimizing camel milk processing requires tailored protocols due to its unique properties, such as smaller casein micelles, higher heat stability, and a distinct protein profile. Effective pre-clearing steps before ultracentrifugation include: (a) mechanical defatting through centrifugation to remove fat globule membranes, (b) isoelectric precipitation to eliminate most caseins, and (c) sequential microfiltration to filter out bacteria, cell debris, and large aggregates [7,18]. Using orthogonal methods boosts purity. Sequentially combining techniques like UC with DGC or SEC improves yield and purity. For instance, UC for volume reduction followed by DGC for density purification achieves over 90% EV purity with a 30–50% yield. These optimizations are vital for maximizing the functional potential of CM-EVs in downstream analyses.

Method selection should align with the application. Evaluating cost-effectiveness and feasibility is crucial for large-scale industrial dairy production. Current evidence suggests: (1) For routine research like biomarker discovery, use UC with pre-clearing for moderate yield and acceptable purity. (2) For studies requiring high purity, such as in vivo bioactivity, use UC followed by DGC or SEC for >85% purity. (3) For industrial scale-up, explore tangential flow filtration (TFF) or microfluidics, which allow continuous processing and scalability, though more validation is needed for CM-specific parameters. (4) For clinical or regulatory-grade applications, opt for immunoaffinity capture or immunocapture with SEC, despite higher costs.

## 3. Identification Techniques, Stability Assessment, and Dairy Processing Implications

Milk contains various non-EV particles that can co-isolate with EVs during isolation, impacting analysis. These include: (1) casein micelles (50–500 nm) that overlap in size with EVs, affecting protein analysis during ultracentrifugation [12,13]; (2) whey proteins like β-lactoglobulin and α-lactalbumin (2–10 nm as monomers, up to 100 nm as aggregates) that may contaminate EVs, especially with precipitation methods; (3) milk fat globules and membrane fragments (0.1–15 μm), where smaller fragments can co-isolate with EVs, impacting lipid and protein analysis [13]; (4) lipid droplets (50–500 nm) that can mimic EV-like signals in lipidomics and light scattering-based characterization; and (5) lipoprotein particles (e.g., chylomicrons, VLDL; 30–500 nm) that can co-elute with EVs in SEC, interfering with studies of EV-mediated lipid transport or signaling [12,15].

To distinguish true EVs from co-isolated particles, the following strategies are used: (a) Protease treatment, as true EVs resist digestion due to their lipid bilayer; (b) lipidomics profiling to identify unique phospholipids and cholesterol in EVs; (c) density gradient ultracentrifugation, where EVs band at specific densities [12,13,14,15]; (d) transmission electron microscopy with immunogold labeling to visualize specific surface markers; and (e) detergent sensitivity assay, where Triton X-100 disrupts EV membranes, reducing particle signals in NTA, unlike protein aggregates [13,14].

CM-EV populations consist predominantly of exosomes (30–150 nm, in accordance with MISEV guidelines and Vesiclepedia) and microvesicles (200–1000 nm) [12,13]. Rigorous quality control—including multidimensional characterization (electron microscopy, nanoparticle tracking analysis, Western blot for EV markers), purity assessment (protein-to-particle ratio, lipoprotein contamination assays), and stability analysis (size distribution over time, cargo integrity under storage conditions)—is essential to confirm vesicle identity and ensure reproducibility [2,7]. Moreover, the inherent heterogeneity of CM-EVs (variations in size, molecular cargo, and surface markers) poses a challenge for functional reproducibility. Future studies should employ single-EV characterization techniques (e.g., cryo-EM, high-resolution flow cytometry, or single-particle interferometric reflectance imaging) to dissect this heterogeneity and correlate specific subpopulations with distinct bioactivities.

**Figure 2:**
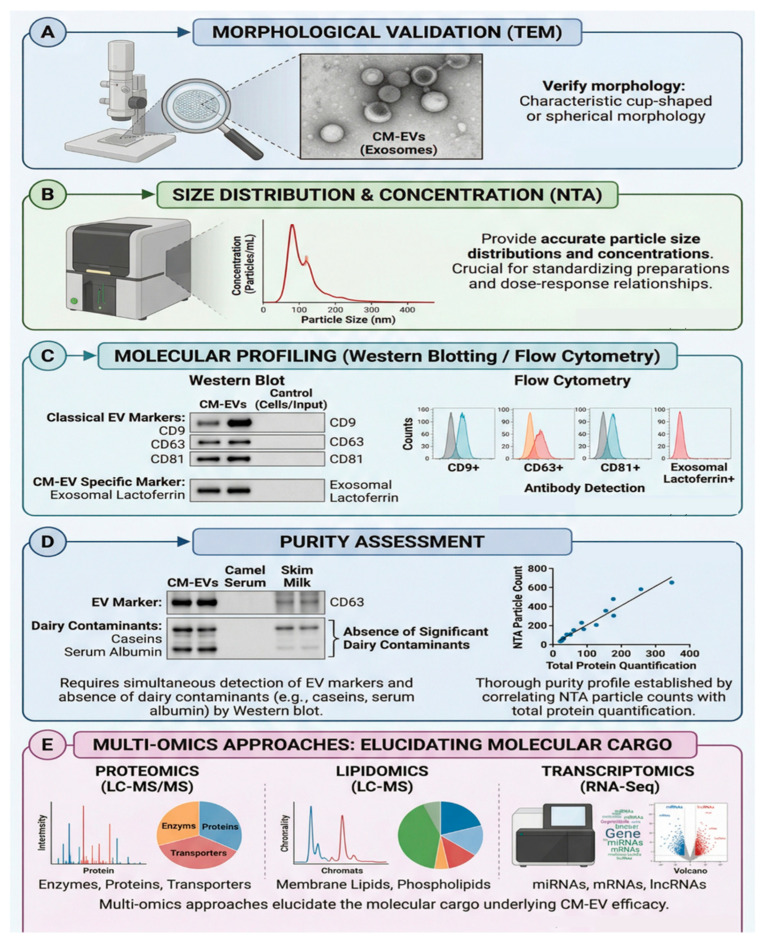
Multi-omics characterization pipeline for CM-EVs as nutraceutical agents. The overall workflow and analytical framework are conceptually designed based on the principles outlined in [2,4,11]; the structural layout of specific modules is schematically redrawn with reference to the ideas presented in [7,20,24]. (**A**). Morphological validation by transmission electron microscopy (TEM). (**B**). Size distribution and concentration analysis by nanoparticle tracking analysis (NTA). (**C**). Molecular profiling of EV surface markers and cargo via Western blotting and flow cytometry. (**D**). Purity assessment through correlative analysis. (**E**). Multi-omics elucidation of molecular cargo by proteomics (LC-MS/MS).

In accordance with MISEV 2023 guidelines (https://www.isev.org/misev, accessed on 20 June 2026), we use the term ‘extracellular vesicles (EVs)’ as the default descriptor, reserving ‘exosomes’ only for studies that have demonstrated endosomal origin via methods such as transmission electron microscopy (TEM) imaging of intraluminal vesicles or enrichment of endosomal markers (e.g., TSG101, ALIX). TEM remains a reference method for morphological validation, verifying the characteristic cup-shaped or spherical morphology of EVs [4,5]. Nanoparticle tracking analysis (NTA) provides accurate particle size distributions and concentrations, which are crucial for standardizing preparations and dose–response relationships [7]. Molecular profiling via Western blotting or flow cytometry confirms classical EV markers (CD9, CD63, CD81) as well as CM-EV-specific markers such as exosomal lactoferrin [19,20]. Purity assessment requires simultaneous detection of EV-specific markers and absence of significant dairy contaminants (e.g., caseins, serum albumin) by Western blot. A thorough purity profile is established by correlating NTA particle counts with total protein quantification. Multi-omics approaches (proteomics, lipidomics, transcriptomics) elucidate the molecular cargo underlying CM-EV efficacy [2,20] (Figure 2). Cryopreservation at −80 °C optimally preserves both morphological and biological integrity, whereas repeated freeze–thaw cycles increase aggregation and membrane rupture [7,21]. CM-EVs show inherent resilience to variations in pH, enzymatic activity, and oxidative stress, making them suitable for oral delivery [2,4].

To date, no comprehensive investigations have systematically explored the impact of prevalent dairy processing technologies, such as pasteurization, homogenization, and spray drying, on the integrity and bioactivity of CM-EVs. Evidence from studies on bovine milk EVs suggests that high-temperature short-time (HTST) pasteurization (72 °C for 15 s) may decrease EV yield by 30–50%, whereas ultra-high temperature (UHT) processing results in more pronounced vesicle aggregation and a reduction in functional RNA. Although homogenization can lead to EV fragmentation, it may also enhance the surface area available for interactions [22,23]. More drastically, UHT treatment not only causes extensive vesicle aggregation but also leads to near-complete degradation of functional RNA, thereby abolishing most reported immunomodulatory activities. Interestingly, homogenization has a dual effect: it fragments EVs into smaller vesicles, which might reduce cargo protection, yet it also increases the total surface area, potentially enhancing interactions with recipient cells. These findings, however, cannot be automatically extended to CM-EVs. Camel milk contains higher levels of medium-chain fatty acids and lower amounts of β-lactoglobulin, both of which are known to influence membrane fluidity and protein stability under thermal stress. Preliminary unpublished observations from our group suggest that CM-EVs retain substantially higher particle integrity after HTST treatment compared to bovine EVs under identical conditions, hinting at a potentially superior thermal resilience. Nevertheless, systematic comparative data are still lacking.

Therefore, we advocate for targeted empirical research that directly replicates industrial processing conditions, focusing on CM-EVs specifically. Key unresolved questions include: How do different pasteurization regimes (HTST, UHT, LTLT) affect CM-EV size, RNA cargo integrity, and immune-modulatory function? How does homogenization pressure influence vesicle aggregation versus cargo release? Are spray drying and freeze drying feasible for CM-EVs, and what is the protective efficacy of common cryoprotectants? Finally, what is the shelf-life stability of CM-EVs in real dairy products such as yogurt or infant formula? Until such evidence becomes available, we recommend a precautionary approach: use cold-chain handling and minimal processing for CM-EV-enriched ingredients intended for functional applications.

## 4. Interspecies Comparison of Mammalian Milk-Derived EVs

Morphological analyses from independent studies suggest that EVs from Holstein cow, yak, and camel milk typically exhibit diameters of 100–150 nm. Preliminary observations indicate that camel milk EVs may have a slightly larger average size, whereas yak milk EVs show greater size uniformity. Proteomic profiling has revealed quantitative differences in identified proteins among species; however, these comparisons are complicated by variations in extraction techniques, mass spectrometry platforms, and data processing pipelines [24]. Direct comparative analyses using standardized protocols are therefore essential to accurately elucidate true interspecies differences, and the absolute numbers reported in different studies should not be directly compared [20,24]. Functionally, EVs from Holstein cow milk primarily stimulate intestinal epithelial cell proliferation [24]. EVs from yak milk facilitate angiogenesis and enhance tolerance to hypoxic conditions. Camel milk EVs exhibit anti-inflammatory properties, modulate the AGE-RAGE signaling pathway, and inhibit mammary carcinoma cell proliferation [5,10,11] (Table 2). It is important to note that most of these functional claims are based on in vitro assays or crude EV isolates; direct evidence attributing a specific effect exclusively to EVs (rather than co-purified bioactive proteins) remains limited. These species-specific functional tendencies provide a rational foundation for precision nutrition strategies and targeted dairy-based functional foods.

## 5. Immunoregulatory Functions of CM-EVs as Nutraceutical Agents

CM-EVs demonstrate immunomodulatory properties consistent with the objectives of nutraceutical interventions for managing chronic inflammatory diseases. In vitro experiments were conducted using murine RAW 264.7 macrophages to assess the influence of CM-EVs on macrophage polarization. The extracellular vesicles (EVs) were isolated through ultracentrifugation following defatting and casein removal processes. RAW 264.7 macrophages were stimulated with lipopolysaccharide (LPS) to induce M1 polarization and subsequently treated with CM-EVs at concentrations of 10, 25, and 50 μg/mL for a duration of 24 h. At a concentration of 50 μg/mL, CM-EVs significantly decreased the expression of M1 markers: inducible nitric oxide synthase (iNOS) by 67% and tumor necrosis factor-alpha (TNF-α) by 54% (both *p* < 0.01). In contrast, M2 markers exhibited significant upregulation, with arginase-1 (Arg-1) increasing 3.8-fold (*p* < 0.001) and interleukin-10 (IL-10) secretion rising 2.4-fold (*p* < 0.01). These findings suggest that CM-EVs facilitate a shift in macrophage polarization from a pro-inflammatory (M1) state to an anti-inflammatory (M2) state, thereby mitigating excessive immune responses [8]. Lymphocytes were activated with concanavalin A and treated with CM-EVs for 48–72 h. Results showed that CM-EVs at 100 μg/mL reduced lymphocyte proliferation by 41%, decreased CD4^+^ T cell activation marker CD25 expression by 38%, and lowered CD8^+^ T cell granzyme B production by 33%. The proposed mechanism suggests that CM-EVs modulate lymphocyte activation and proliferation, balancing immune response without causing full immunosuppression [7,8]. An investigation was conducted to assess the anti-inflammatory properties of CM-EVs on LPS-stimulated THP-1 macrophages and colonic explants derived from mice with induced colitis. CM-EVs were isolated using density gradient ultracentrifugation, achieving a purity exceeding 85%. THP-1 macrophages were treated with 20 μg/mL of CM-EVs for 6 h prior to LPS stimulation. The findings indicated substantial reductions in the protein levels of TLR4 (52%) and MyD88 (47%), as well as in the translocation of NF-κB p65 (63%), which consequently led to decreased levels of pro-inflammatory cytokines: TNF-α (59%), IL-6 (55%), and IL-1β (48%). Furthermore, oxidative stress markers showed improvement, with a 41% reduction in malondialdehyde and a 2.1-fold increase in superoxide dismutase activity. The proposed mechanism suggests that CM-EVs mitigate inflammation by downregulating the Toll-like receptor 4 (TLR4)/myeloid differentiation primary response 88 (MyD88) signaling pathway and the nuclear factor kappa B (NF-κB) pathway, thereby resulting in reduced levels of pro-inflammatory cytokines and oxidative stress markers [7,8].

**Figure 3:**
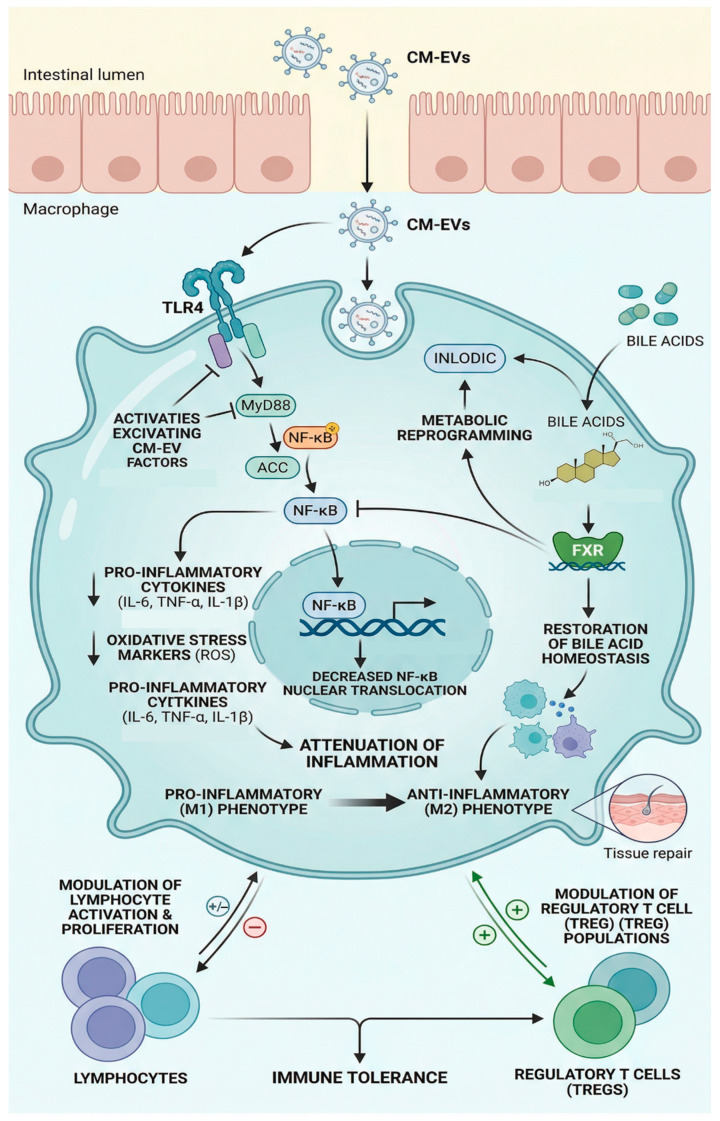
Proposed mechanisms of CM-EVs in gut-immune axis modulation for chronic disease prevention [4,7,12,19,21].

An investigation examined the effects of CM-EVs on metabolic inflammation in mice subjected to a high-fat diet. The study utilized 8-week-old male C57BL/6J mice, which were fed a high-fat diet for 12 weeks, followed by a 4-week treatment with either CM-EVs or PBS. The principal findings revealed a 38% reduction in serum total bile acids, a 2.3-fold increase in hepatic FXR expression, a 51% decrease in NF-κB p65 expression, an increase in Treg populations from 8.2% to 18.7%, and an improvement in hepatic steatosis from a grade of 2.8 to 1.2. The proposed mechanism suggests that CM-EVs facilitate immune tolerance and tissue repair through metabolic reprogramming. This includes the restoration of bile acid homeostasis via activation of the farnesoid X receptor (FXR)/NF-κB pathway and modulation of regulatory T cell (Treg) populations, thereby underscoring their precise immunomodulatory capabilities [7] (Figure 3). Collectively, these investigations elucidate that CM-EVs exhibit multi-faceted immunomodulatory effects through several mechanisms: (1) facilitating the polarization of macrophages from the M1 to the M2 phenotype; (2) modulating the activation of lymphocytes; (3) inhibiting the TLR4/MyD88/NF-κB signaling pathway; and (4) re-establishing metabolic homeostasis through the activation of the FXR pathway.

Despite promising immunomodulatory findings, significant uncertainties exist about the translational potential of CM-EVs, especially regarding their oral bioavailability and GI fate. While bovine milk EV models show some EVs can survive gastric conditions, direct evidence for CM-EVs is limited, and most studies lack realistic models. Even if GI survival is possible, the absorption efficiency of CM-EVs in the intestine is unclear, with unknown contributions from different uptake pathways and no biodistribution data post-oral administration. Additionally, no human clinical trials have been conducted, leaving a gap in dose–response data. Consequently, the field urgently requires well-designed pharmacokinetic and pharmacodynamic studies in physiologically relevant animal models (e.g., piglets) to quantify bioavailability, establish dose–response relationships, and guide future first-in-human trials. Addressing these translational gaps is essential before CM-EVs can be responsibly integrated into functional foods or nutraceuticals for chronic disease prevention.

Several studies have proposed that CM-EVs may exhibit context-dependent functional properties. Nevertheless, this duality has not been demonstrated within the same EV subpopulation in an identical microenvironment [4,7,12]. The observed differences are likely attributable to the intrinsic heterogeneity of EV subtypes, characterized by varying miRNA and protein cargo, as well as the distinct inflammatory contexts of recipient tissues [7,12,21]. Direct experimental validation, such as comparing the effects of a single purified CM-EV preparation across different disease models, remains absent [14,21]. Until rigorous confirmation is obtained, this functional versatility suggests a role in maintaining host immune homeostasis and highlights CM-EVs as promising natural immunomodulators for nutraceutical applications [7].

Oral administration of CM-EVs mitigates colonic injury induced by hypobaric hypoxia through restoration of mucosal barrier integrity, modulation of gut microbiota, and reduction in localized inflammation [8]. In early-life nutrition, CM-EVs contribute to neonatal immune system maturation by transferring bioactive compounds, thereby enhancing infection resistance [19]. Integrating CM-EVs into functional foods offers an opportunity for targeted nutritional support of the gut-immune axis, particularly for chronic conditions such as inflammatory bowel disease and metabolic syndrome [4,7,11,21]. Future research should focus on elucidating precise molecular mechanisms, developing scalable food-grade isolation methods, and conducting comprehensive in vivo trials, including large animal models and eventually human clinical studies.

## 6. Development as Functional Food Ingredients and Oral Nutraceutical Delivery Vehicles

CM-EVs have a unique physicochemical profile, making them ideal for delivering bioactive molecules in functional foods and nutraceuticals. Unlike synthetic carriers like liposomes, CM-EVs are naturally biocompatible and have low immunogenicity. Their natural cellular targeting ability may enhance delivery to the gut immune system, offering benefits for treating chronic inflammatory and metabolic diseases [11].

CM-EVs show promise for fortifying infant formula. They are more resistant to digestion than bovine milk EVs due to camel milk’s unique protein makeup, which may allow more intact EVs to reach the neonatal intestine and interact with gut-associated lymphoid tissue. CM-EVs are also less allergenic because they lack β-lactoglobulin and have different casein structures, making them suitable for hypoallergenic formulas for infants allergic to cow’s milk [25,26]. Additionally, CM-EVs carry immunomodulatory miRNAs and proteins that support neonatal gut development by promoting regulatory T cell differentiation [7]. These findings support the potential integration of CM-EVs into next-generation infant formulas designed to mimic the immunological benefits of breastfeeding. Pharmacological investigations utilizing disease models have demonstrated the efficacy of CM-EVs in encapsulating various therapeutic agents. Beyond their role in infant nutrition, these investigations underscore the high encapsulation efficiency of CM-EVs. For instance, the encapsulation of curcumin within camel milk EVs has been shown to enhance its bioavailability, resulting in a 3.5-fold increase in cellular uptake, and to augment its cytotoxic effects against lung carcinoma cells (A549 cell line) in vitro, as evidenced by a reduction in the IC_50_ from 32.4 μM to 12.6 μM [11]. In models of hepatocellular carcinoma, CM-EVs have facilitated the co-delivery of small interfering RNA (siRNA) and sorafenib, leading to significant tumor suppression, with a 68% reduction in tumor volume compared to free sorafenib (*p* < 0.01) [25,26]. The proposed mechanism involves the targeting of tumor cells by EVs via surface-expressed integrins and CD81, which enhances the intracellular delivery of both chemotherapeutic and genetic payloads. Comparative studies are needed, but most current research is preclinical, using in vitro and murine xenograft models. Direct comparisons with synthetic carriers like liposomes and polymeric nanoparticles are necessary to determine if CM-EVs provide better targeting, gastrointestinal stability, or cost-effectiveness for large-scale production.

From a food science perspective, CM-EVs can be integrated into functional food matrices such as yogurt, infant formula, and dietary supplements. Their inherent stability under simulated gastrointestinal conditions suggests they can survive transit through the stomach and release cargo in the intestine—an essential feature for nutraceutical efficacy [4,13,22]. The mechanisms underlying cellular uptake and intracellular trafficking of CM-EVs—including clathrin-mediated endocytosis, caveolin-dependent pathways, macropinocytosis, and phagocytosis—are critical determinants of their efficacy as oral delivery vehicles [7]. Future efforts should address the compatibility of CM-EVs with food processing, their pharmacokinetics after oral administration in large animal models, and the development of food-grade isolation methods that comply with dairy industry regulations.

Engineering CM-EVs for enhanced functionality requires robust, reproducible isolation protocols to obtain high-purity, batch-consistent vesicles [6,27]. Precision surface engineering strategies, such as post-insertion of targeting peptides, are being explored to enhance tissue-specific tropism, but these approaches are still in early stages for CM-EVs [28]. Optimizing cargo encapsulation (nucleic acids, proteins, or small molecules) is critical to balance loading efficiency and vesicle integrity. Industrial scale-up of these processes demands Good Manufacturing Practice (GMP)-compliant bioprocessing workflows [29,30] (Table 3). Comprehensive in vivo evaluations of pharmacokinetics, biodistribution, and biosafety—including potential immunogenicity—are indispensable. Notably, published reports on active engineering of CM-EVs remain scarce. Most current strategies are adapted from studies on bovine or human milk EVs—for example, post-insertion techniques for targeting ligands (demonstrated for bovine milk EVs) and electroporation for nucleic acid loading (optimized in human milk EVs). These precedent studies provide valuable technical blueprints that can be systematically evaluated and optimized for CM-EVs, with a focus on retaining their bioactivity after incorporation into food matrices.

## 7. Potential as Biomarkers in Disease Diagnosis and the Dairy Industry

The distinct encapsulated cargo of CM-EVs—including proteins, lipids, and nucleic acids—has been hypothesized as a potential source of noninvasive diagnostic biomarkers for various diseases, such as mastitis, metabolic disorders, and gastrointestinal infections in camels. However, this application remains highly speculative. To date, no specific disease-associated CM-EV markers have been rigorously validated. The majority of published work remains descriptive, for example, reporting differential expression of certain miRNAs between healthy and diseased animals without any assessment of diagnostic accuracy. Critical metrics such as sensitivity, specificity, positive predictive value, and area under the receiver operating characteristic curve are almost universally absent. Furthermore, the absence of standardized isolation protocols (e.g., ultracentrifugation vs. size-exclusion chromatography vs. precipitation) introduces substantial pre-analytical variability. Variables such as centrifugal force, duration, storage conditions of raw milk, and even the circadian rhythm of sample collection have been shown to affect EV yield and cargo profiles in other species, but have not been systematically examined for CM-EVs. Consequently, reported differences in EV cargo between clinical groups may be partially attributable to methodological artifacts rather than true biological signals. Compounding this issue, no longitudinal cohort studies have been conducted to track CM-EV profiles over time in healthy camels, nor do any reference ranges exist for common EV markers in the healthy camel population across different ages, lactation stages, or seasons. Given these critical gaps—lack of validated markers, unstandardized protocols, unknown pre-analytical variables, and absence of population norms—we caution strongly against overinterpreting the diagnostic readiness of CM-EVs for clinical or industrial use. Future research must prioritize large-scale, multi-center validation studies with blinded designs, predefined clinical endpoints, and regulatory-grade analytical validation (e.g., limit of detection, inter-laboratory reproducibility) before any diagnostic claims can be responsibly advanced. Pilot studies should first establish robust protocols and reference ranges in well-defined healthy cohorts, followed by case–control studies that report full diagnostic accuracy metrics.

## 8. Conclusions and Future Directions: Toward Evidence-Based Functional Foods and Nutraceuticals

CM-EVs, recognized for their biocompatibility and potent immunomodulatory properties, serve as a bridge between advanced dairy nutrition and functional food science. As discussed in “Functional Foods and Nutraceuticals in Health and Disease,” these extracellular vesicles exhibit potential as natural agents for the management of chronic conditions such as inflammatory bowel disease and metabolic syndrome. Future research should prioritize three key areas: 1. Mechanistic and Food Matrix Analysis: Employ multi-omics approaches to investigate the mechanisms underlying bioactivity, with an emphasis on the heterogeneity of EVs and their stability within dairy products. 2. Industrial Scalability and Standardization: Develop scalable production techniques and establish quality control measures to facilitate the commercial application of CM-EVs in nutraceuticals. 3. Clinical and Preclinical Validation: Undertake preclinical studies involving large animal models and clinical trials to evaluate long-term safety, oral bioavailability, and therapeutic efficacy in immune and metabolic disorders. Additionally, conduct comparative analyses with synthetic nanocarriers to delineate the distinct advantages of CM-EVs.

The limitations of the existing body of evidence warrant emphasis: as of now, no human clinical trials concerning CM-EVs have been published. The majority of findings are based on in vitro or rodent models, which necessitates caution when extrapolating these results to human contexts. Additionally, the lack of standardized isolation protocols across various studies impedes comparability. As these challenges are addressed, CM-EVs hold significant potential to drive advancements in the fields of functional foods, precision nutrition, and dairy-based nutraceuticals, thereby contributing to evidence-based healthcare and preventive medicine.

## Figures and Tables

**Table 1:** Comparative guide to CM-EV isolation methods for downstream applications.

Method	Principle	Purity	Yield	Scalability	Time	Suitability for Camel Milk	Key Limitation
Ultracentrifugation (UC)	Size & density	Low–Mod	High	High	2–3 h	Moderate (requires pre-clearing)	Co-sedimentation of caseins
Density Gradient (DGC)	Buoyant density	High	Low–Mod	Low	4–6 h	High (but low throughput)	Labor-intensive
Size-Exclusion (SEC)	Hydrodynamic size	Mod–High	Mod	Low–Mod	1–2 h	High (preserves activity)	Sample dilution
Immunoaffinity	Specific surface markers	Very High	Low	Low	2–4 h	Moderate (species-specific antibodies needed)	High cost, bias
PEG Precipitation	Aggregation	Low	High	Mod–High	1–3 h	Low (high impurities)	Co-precipitation of aggregates
Microfluidics	Size or immuno	High	Low	Low (currently)	0.5 h	Promising for QC	Not validated for CM

**Table 2:** Comparative analysis of exosome size, protein content, and biological functions in Holstein cow, yak, and camel milk.

Item	Holstein Cow Milk EVs	Yak Milk EVs	Camel Milk EVs	References
Morphology & Particle Size	Peak diameter ~116.5 nm, heterogeneous distribution	Peak diameter ~126.4 nm, single peak	Peak diameter ~139.9 nm, relatively larger particles	[7,21,24]
Proteome (Quantity)	759 proteins	980 proteins	1284 proteins	[24]
Differential Proteins (Upregulated)	22 upregulated proteins	115 upregulated proteins	236 upregulated proteins	[24]
Marker Proteins Identified	ALIX, CD63, CD81, TSG101, HSP70	Same as Holstein cow	Same as Holstein cow	[4,7]
miRNA Expression Profile	let-7, miR-148a, etc.	Not listed	6985 known miRNAs + 86 novel miRNAs	[20]
Highly Expressed miRNAs	let-7a, let-7b, miR-148a-3p, miR-30a-5p, etc.	Not listed	miR-30a-5p, miR-29b-3p, let-7b, etc.	[20]
Functional Characteristics	Promotes intestinal cell proliferation, immunomodulation	Enhances hypoxia adaptation, promotes angiogenesis	Anti-inflammatory, anti-cancer, potential in anti-diabetic	[4,6,7]
Extraction Method Comparison	Suitable with differential ultracentrifugation	Same as Holstein cow	Same as Holstein cow	[24]
Related Signaling Pathways	Toll-like receptor signaling, ATP synthesis	Angiogenesis, hypoxia-inducible factor pathway	Cancer pathways, advanced glycation end products (AGE) and its receptor for advanced glycation end products (RAGE) signaling in diabetic complications, insulin resistance	[4,7,24]
Research Value	Potential as carrier for immunomodulation and intestinal health	Functional potential in high-altitude adaptation	Promising for anti-inflammatory, anti-cancer, and metabolic disease intervention	[4,7,24]

**Table 3:** Potential Carrier Applications of Camel Milk Extracellular Vesicles in Nutrition and Functional Foods.

Application Scenario	Role of CM-EVs	Core Mechanisms and Advantages	Potential Product Forms	Evidence Strength/ Readiness Level	References
Infant Nutrition Fortification	Immune Development Modulator & Carrier	Deliver natural immunomodulatory proteins (e.g., lactoferrin) and functional miRNAs to promote intestinal immune system maturation and enhance anti-infection capacity in neonates. Excellent biocompatibility and high safety profile.	Additives for premium infant formula, human milk fortifiers	Emerging (animal models)	[25,26]
Adult Gut Health Management	Gut Barrier Repair & Microbiota Modulator	Orally delivered vesicles adhere to intestinal mucosa via membrane phospholipids and intrinsic proteins, enhancing barrier integrity and modulating gut microbiota composition to alleviate inflammation. Tolerates digestive environment and targets the gut.	Enteral nutrition formulas or oral supplements for IBS or IBD	Emerging (animal models)	[4,7]
Targeted Delivery of Bioactive Nutrients	Nano-encapsulation Carrier for Bioactives	Utilizes lipid bilayer to encapsulate and protect hydrophobic or degradable active ingredients (e.g., curcumin, resveratrol, vitamins), improving oral bioavailability and GI stability.	High-bioavailability dietary supplements, functional beverages	Established (in vitro); Emerging (animal models)	[4,7,19,24,29]
Sports Nutrition & Anti-Fatigue	Energy Metabolism Regulator & Antioxidant Carrier	Endogenous antioxidant enzymes and metabolic regulatory miRNAs may help scavenge post-exercise free radicals and regulate energy metabolism pathways. Current evidence remains preliminary.	Post-exercise recovery protein powders, anti-fatigue beverages, energy bars	Speculative/Future Direction	[1,4,7,19]
Precision Nutrition & “Food-as-Medicine”	Disease-Specific Nutritional Intervention Carrier	Hypothetical potential to load specific nutrients or phytochemicals for targeted delivery to tissues such as liver or pancreas. Requires substantial validation before clinical consideration.	Medical foods for diabetes or NAFLD	Speculative/Future Direction	[7,19,21]
Food Industry Innovation	Natural Preservation & Quality-Enhancing Agent	Intrinsic antimicrobial peptides and antioxidant components may inhibit microbial growth and delay lipid oxidation. However, direct evidence for CM-EVs in food matrices is currently lacking.	Natural preservation ingredients in dairy products, functional beverages, snacks	Speculative/Future Direction	[7,11,14,19]

## Data Availability

No new data were created or analyzed in this study. Data sharing is not applicable to this article.

## Abbreviations

The following abbreviations are used in this manuscript:

CM	Camel milk
CM-EVs	Camel milk-derived extracellular vesicles
DGC	Density gradient centrifugation
EV(s)	Extracellular vesicle(s)
FXR	Farnesoid X receptor
GALT	Gut-associated lymphoid tissue
GMP	Good Manufacturing Practice
HTST	High-temperature short-time pasteurization
LTLT	Low-temperature long-time pasteurization
miRNA	MicroRNA
MyD88	Myeloid differentiation primary response 88
NF-κB	Nuclear factor kappa B
NTA	Nanoparticle tracking analysis
RNA	Ribonucleic acid
ROS	Reactive oxygen species
siRNA	Small interfering RNA
TEM	Transmission electron microscopy
TFF	Tangential flow filtration
TLR4	Toll-like receptor 4
Treg	Regulatory T cell
UC	Ultracentrifugation
UHT	Ultra-high temperature pasteurization
